# Exploration of priority actions for strengthening the role of nurses in achieving universal health coverage

**DOI:** 10.1590/1518-8345.1696.2819

**Published:** 2017-01-30

**Authors:** Rowaida Al Maaitah, Raeda Fawzi AbuAlRub

**Affiliations:** 1 PhD, Professor, Jordan University of Science and Technology, Irbid, Jordan.

**Keywords:** Nursing, Advanced Practice Nursing, Human Resources, Health Policy, Education

## Abstract

**Objective:**

to explore priority actions for strengthening the role of Advanced Practice Nurses (APNs) towards the achievement of Universal Health Converge (UHC) as perceived by health key informants in Jordan.

**Methods:**

an exploratory qualitative design, using a semi-structured survey, was utilized. A purposive sample of seventeen key informants from various nursing and health care sectors was recruited for the purpose of the study. Content analysis utilizing the five-stage framework approach was used for data analysis.

**Results:**

the findings revealed that policy and regulation, nursing education, research, and workforce were identified as the main elements that influence the role of APNs in contributing to the achievement of UHC. Priority actions were identified by the participants for the main four elements.

**Conclusion:**

study findings confirm the need to strengthen the role of APNs to achieve UHC through a major transformation in nursing education, practice, research, leadership, and regulatory system. Nurses should unite to come up with solid nursing competencies related to APNs, PHC, UHC, leadership and policy making to strengthen their position as main actors in influencing the health care system and evidence creation.

## Introduction

The dynamic nature of the health care sector, coupled with complex challenges and continuous health care reforms, have typically imposed changes in the nature and scope of health care professionals’ roles, especially nurses. Recently, during the September 2015 United Nations (UN) summit, global leaders adopted a very challenging global development agenda, which included seventeen Sustainable Development Goals (SDG) and reaffirmed their commitment to Universal Health Coverage (UHC) as one of the major targets of the global health goal^1^.The global health goal of the SDG aims to ensure healthy lives and promote well-being for all. The global health goal has eight targets, including the UHC^1^ ^-^ ^2^.

UHC promotes healthier lives for all ages, and this will not be attained unless there is a true investment in health care systems and the health workforce. Evidence suggests investments in health systems are key components to improved health outcomes^2^ ^-^ ^3^. UHC does not only remove the accessibility and quality barriers to health care, but the financial barriers as well to everyone including the poor. In 2010, the World Health Report emphasized the disastrous outcome of health care cost in which the out-of-pocket spending had pulled down around 100 million worldwide below the poverty line^3^. Evidence suggests that effective provision of affordable, acceptable, high- quality health care services leads to the improvement of a population’s health, especially for vulnerable individuals and communities^4^. The causal analyses from 153 nations revealed that broader health coverage provided better access to necessary health care services and improved the health of the population, with considerable gains to the impoverished^5^.

In an attempt to accelerate progress towards the global health agenda including UHC, a global strategy on human resources for health has been developed by the WHO^6^. Access to essential, quality health care services depends mainly on the key determinant of the supply of health labor, which is the education and training of health care workers. The nursing profession has shown a strong interest in UHC.UHC has clearly articulated the vision of the 2016-2020 Strategic Directions for Nursing and Midwifery (SDNM) “to ensure that the nursing and midwifery workforce contributes to UHC and the Sustainable Development Agenda, by ensuring equitable access to skilled and motivated nursing and midwifery workforces within performing and responsive health systems”^7^.

According to the World Health Organization Regional Office for the Eastern Mediterranean Region (WHO EMRO), one of the main key elements of strengthening health care systems is health workforce planning, production, training and retention which requires the development of well-articulated, well-prepared, and well-managed health care workforce with the appropriate skills mix to meet the needs of their countries^8^.There are a number of challenges facing medical and nursing education in the WHO EMRO, including increased investment on tertiary care, on the expense of prevention and health promotion, as well as the concentrated training and preparation of students within the hospital walls with less exposure to community and primary health care (PHC) settings^9^. Similar problems were reported by the summary report on the regional nursing forum regarding the future of nursing and midwifery in the EMRO, which identified education as one of the main challenges for nursing and midwifery in addition to workforce, practice and service development, regulation, governance, and information systems^10^.

Jordan has one of the most modern health care infrastructures in the Middle East, and it has been ranked the first in the region for medical tourism^11^. Despite the improvement of the health indicators in Jordan, the rates of chronic illnesses are increasing. The mortality rates from Non Communicable Disease (NCD) in Jordan was 727 per 100,000 population in 2008 compared to 573 per 100,000 population of the global NCD mortality rate in the same year^11^. In addition, the economic growth remains a challenge which has been hindered by global economic depression; the unstable political status in the region; the influx of refugees throughout the last 15 years, which has made great pressure on the education and health sectors especially; the scarcity of natural resources and high stock of external debt.

Unfortunately, the health care sector has invested heavily in curative and tertiary care at the expense of the primary health care, despite the fact that Jordan has a widespread network of PHC centers. The amount of spending on PHC and prevention services from the size of public sector spending is far behind the share of the secondary health care services which amount to more than 72%, compared to 16.4% for PHC and prevention services^11^. UHC is one of the main goals of the National Strategy for the Health Sector in Jordan (2015-2019) and it faces different challenges^11^. In addition, challenges of the health workforce do exist in Jordan and influence the efficiency and effectiveness of service delivery, especially with regards to the PHC sector and UHC^11^ ^-^ ^13^.

In Jordan, the baccalaureate degree in nursing is offered by 15 university programs in addition to 13 master degree programs in 6 universities and one national doctorate program^12^. Associate degrees in nursing and midwifery are offered by 26 universities and 4 colleges, following the termination of the diploma nursing programs in 2002^12^ ^-^ ^13^. While the previous reform in nursing education in Jordan has enhanced the status of the nursing profession, further improvement of the sector is required so as not to jeopardize the long-term quality of nurse graduates^12^ ^-^ ^13^. Still more striking is the incongruence between the traditional nursing education system, with the contemporary challenges facing the health care sector, including the changing health needs of the population. Hence, the gap among nursing education, the practice sector, and patients’ needs is widening. In addition, the absence of regulations to improve the advanced nursing role as well as the lack of clarity nursing role and job descriptions have marked the nursing profession with lack of autonomy and decision-making power^10^ ^,^ ^12^ ^-^ ^13^.

According to the International Council of Nurses (ICN), Advanced Practice Nurse (APN)/ a Nurse Practitioner is defined as “a registered nurse who has acquired the expert knowledge base, complex decision-making skills and clinical competencies for expanded practice, the characteristics of which are shaped by the context and/or country in which s/he is credentialed to practice. A Master’s degree is recommended for entry level”^14^.

Many countries of the EMRO are still struggling to introduce the role of APNs, and Jordan is no exception^10^. Laws in Jordan are behind in the area of advanced practice nursing, regardless of the high number of nurse specialists graduates. One of the main barriers is the dominance of the medical profession; in addition to the lack of awareness of policy makers, and health professionals about the importance of APNs in promoting the population’s health, and achieving the country’s health agenda^10^ ^,^ ^12^ ^-^ ^13^. Given the global shortage of the health workforce, most countries are searching for solutions to improve their health care systems. One solution is to utilize APNs to provide PHC autonomously and independently, through the performance of assessments and diagnoses, ordering diagnostic and laboratory tests, as well as prescribing medications and offering treatments. In addition, APNs could monitor patients’ adherence to medical plans, and offer both counseling and education for non-communicable disease prevention^15^.

A meta-analysis of 11 trials and 23 observational studies revealed patients were more satisfied by services provided by nurse practitioners, than those provided by physicians. In addition, the results asserted there were no detected differences in patient health status, or the number of prescriptions and/or consultations^16^.Another systematic review of 37 studies, over a span of 18 years (1990-2008), on all types of APNs, revealed a significant amount of evidence to support equality on measures of the following outcomes, when comparing nurse practitioners and physicians: mortality, functional status, patient satisfaction, blood pressure control, self-reported patient perception of health, patient glucose control, and utilization rates of emergency department/urgent care^17^.The abovementioned comprehensive systematic reviews asserted the significant role of APNs, in regards to outcomes of patients and clients, which will contribute to the global health agenda, including UHC.

There continues to be a shortage of literature on the roles of APNs in improving the outcomes of health care including UHC. There is also a lack in research reflecting the views of nurse leaders, key health informants, and policy makers for strengthening the role of APNs to achieve UHC. Therefore, the purpose of this study was to investigate priority actions for strengthening the role of APNs towards the achievement of UHC, as perceived by key health informants in Jordan. The results of this study will inform health policy decisions and nurse leaders regarding main issues that demands priority actions to set the stage for advancing nurses’ roles and scope of practice, thereby contributing to the national health agenda, including the UHC.

## Methods

### Design

This exploratory, qualitative study uses a semi-structured survey to elucidate the views of key health informants regarding priority actions for strengthening the role of APNs, towards the achievement of UHC. Because of the dearth of research on this issue in Jordan and the Middle Eastern countries, this design will help researchers and policy makers in understanding the major issues, as perceived by experts of key leaders.

### Sample and Setting

A purposive sample of seventeen key health informants from various nursing and health care sectors in Jordan were selected for the purpose of this qualitative study. Key informants included experts and policy makers from the education sector (public and private nursing schools), the Ministry of Health, nursing associations, health and nursing councils, and hospitals. The Age of participants ranged from 40-65 years. All are specialized and have graduate degrees. Most with doctoral degrees in Nursing (n = 10) and Medical field (n = 2), 5 with master degree in nursing. The participants were 1. policy makers in nursing at the academic sector including deans, vice deans and chairpersons of community health departments (n = 12) participants; an 2. policy makers in professional organizations including the president of the nursing association, secretary general of the Jordanian Nursing Council (the nursing regulatory body), the director of nursing at the Ministry of health, the secretary general of the Higher Health Council who is also the secretary general of the medical council, and the director of planning and development at the Higher health council.

### Data Collection and Ethical Consideration

Approval from the Institutional Review Board, of the affiliated university, was granted prior to the implementation of the study. The researchers approached key informants via phone in order to seek their approval for participation. The purpose of the study was explicated for each person, and questionnaires with cover letters, were sent via email after securing their approval of participation. The cover letter included information about the purpose and the importance of the participants’ responses in this study, the first of its kind in Jordan, on identifying priority actions for strengthening the role of APNs towards the achievement of UHC. The cover letter also included information about voluntary participation and confidentiality of responses. The participants were also assured results will be reported in aggregates, and all personal information will remain confidential. Participants were also given the choice to send their responses via email, fax, and/or mail. The participants’ responses were coded without identities, categorized according to questions, and entered into a spread sheet for thematic/ content analysis.

The survey questions were developed based on the authors’ experiences, and from the synthesis of the literature which addressed UHC and nursing practice. The survey questions were examined and revised by a panel of nursing experts who reviewed and verified the relevance and scope of the questions to the study’s purpose.

### Data Analysis

Content analysis utilizing the five stage ‘framework approach’(18) was used for data analysis. The five stages of the framework included familiarization, identification of thematic framework, indexing of the transcripts, abstraction, and synthesis. The stages were applied as follows: 1. the researchers looked at the data and identified key elements and repeated themes; 2. the identified thematic framework was based on survey questions, purpose of the study, and themes that appeared during the familiarization stage; 3. the index or the identified themes were applied to all data; 4. data were charted to appropriate themes; and summaries of views were formed; 5. the researchers examined the summaries and looked for associations between themes to help explain the results.

## Results

**Figure 1:**
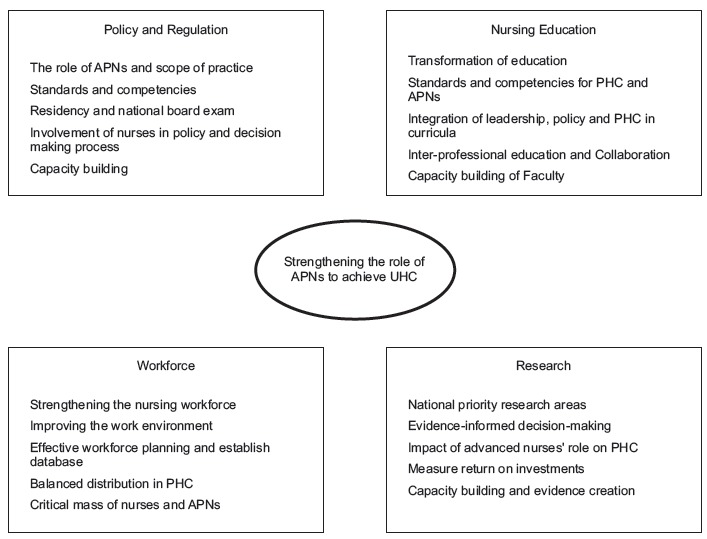
Priority actions to strengthening the role of APNs to achieve UHC

The findings revealed the following themes regarding priority actions for strengthening the role of APNs towards the achievement of UHC: policy and regulation, nursing education, workforce, and research as shown in Figure 1.

### Policy and Regulation

Five issues were emphasized by key informants pertaining to priority actions for the theme of policy and regulation: expansion of the role of APNs and scope of practice; development of standards and competencies for nursing education, practice, leadership and APNs; establishment of residency and national board exam; involvement of nurses in the policy and decision-making process and capacity building of nurses and nurse leaders indifferent sectors and levels.

Most key informants emphasized that expanding the role and scope of the nursing practice of APNs requires solid regulatory framework, in order to ensure and sustain a legal status for expanding the role of APNs in PHC settings to achieve UHC:

*Develop policies that emphasize the need for providing PHC as an essential component of the health care system and enhance the role of advanced prepared nurses as essential health care providers for achieving UHC.*

Another participant elaborated on the role of APNs:

*The role of the APN should be made clear to nurse specialists themselves as well as health institutions. The job description should emphasize the five main roles of the APNs which include: evidence-based direct care, teaching and counseling, research, leadership/management and ethical decision-making. Development of APNs’ leadership is important. Nurse leaders/researchers need to provide evidence that APNs can make a difference in the health care system.*

The low level of nurses’ involvement in the policy and decision-making process was also a main concern voiced by most key informants. Increasing visibility of nurses in policy and decision- making, as well as national boards and committees, will enable nurses to effectively impact the health care system and the role of APNs:

*It is important to develop a national strategy by nurse leaders that aims at empowering and preparing competent nurses as policy makers who have a control over their own education and practice; enable nurses to utilize decision-making process at the institutional and professional levels; and have a policy and enforce a policy at the national level to increase the involvement of nurses at the different national committees that are responsible for policy development, implementation and evaluation.*

Further skills are required for nurses as pointed out by another key informant:

*Nurse leaders need to support building up the management, leadership, policy and decision making capabilities and skills of nurses to strengthen the delivery of services; and develop evidence-based education and practice.*

Moreover, the participants indicated that the regulatory body of nursing should help in identifying and dealing with national priority areas related to enhancing the nursing profession and the health care system in Jordan. Establishing a residency program, and a national board exam for Jordanian nurses were among the main issues discussed by the participants to ensure the preparation of highly competent nurses with various advanced specialties. According to one of the key informants:

*We need to implement nurse residency programs. The Jordanian Nursing Council along with all educational institutions should provide full support to this important initiative and enhance graduate nurses’ progress and successful completion of the future residency programs.*

Capacity building in terms of developing leadership and policy making skills as well as competencies of APNs was also addressed by most key informants:

*….enhance and raise awareness related to the advanced nursing practice in Jordan and how it will contribute to the achievement of UHC and access to health.*

### Nursing Education

Several issues emerged concerning nursing education in promoting or strengthening the role of nurses so as to contribute to UHC: transformation of education; establishment of standards and competencies for PHC and APNs; integration and mainstreaming of leadership, policy and PHC at all levels of education; promotion of inter-professional education, collaboration, and capacity building of faculty members. Almost all key informants asserted the pressing need for transforming nursing education. They clarified that profound changes are needed to improve quality of nursing education, adopt competency-based curriculum and enhance the preparation of APNs.

Enhancing the quality of nursing education to produce competent, highly-qualified nurses who are able to meet national health changing needs was voiced by most key informants. The limited focus on PHC in nursing curricula was indeed a major issue which was brought up by almost all key informants. Key informants emphasized the need to strengthen the status and quality of PHC in the nursing profession and health care programs as a basic step towards the establishment of core competencies for PHC to achieve UHC. One of the key informants summarizes this concern as follows:

*Nursing schools need to design and implement curricula that take into account teaching and training nursing students from holistic perspectives, with an emphasis on health promotion, disease prevention, while providing care to patients, families and community in the different health care settings. The relevance of quantity and quality of the nursing and midwifery workforce is essential to meet the local and national health changing needs.*

Integrating leadership and health policy development skills in nursing curricula at different levels of education was referred by key informants as an essential tool for preparing future nurses as change agents with strong voices in the board rooms of decision making. One of the key informants stressed this as:

*It is important to integrate comprehensive policy courses in nursing curricula at all levels, conduct field visits to policy making bodies in Jordan, and invite experts from policy making bodies to lecture on certain policy topics.*

According to key informants, promoting the interdisciplinary and inter-professional practice and establishment of collaboration models between nursing and other disciplines would enhance the attention to national issues which affect the health care system, including UHC. They stressed the importance of developing tools to improve inter-professional partnerships at the inter-sectoral level among health care services, professional associations, health organizations, and educational institutions:

*Schools of nursing, in collaboration with other health professional schools, should design and implement early and continuous inter-professional collaboration through joint classroom and clinical training opportunities.*

*We will need to design evidence-based learning experiences that prepare nurses to work in inter-professional health care teams and manage care transitions among the different settings.*

*Nursing institutions need to be responsible for providing competency-based education that responds to community needs and issues of UHC as well as strengthening nursing education that leads to health promotion and disease prevention. In addition, nursing education institutions should lead national and international efforts for improving education and practice standards. Nursing curricula in Jordan need to move from the traditional approach to competency and evidence-based models of education.*

Building nurse educators’ capacities was emphasized by participants as integral component in transforming traditional nursing education into competency and evidenced based learning:

*I believe we need to focus on building the capacity of faculty members; this is a key factor in transforming nursing education. It is also important to focus on academic preparation in nursing education and faculty development programs to help novice educators develop their teaching skills in interactive learning environments.*

### Workforce

Issues discussed by participants concerning the theme of workforce were: strengthening and empowering the nursing workforce and investing in their full potentials, improving the work environment including PHC settings, ensuring effective nursing workforce planning, including establishment of a database for the nursing workforce, maintaining an effective balanced distribution of nurses in PHC and the creation of critical mass of general nurses and APNs.

Key informants of the present study voiced their opinions regarding the importance of empowering and strengthening nursing workforce as well as enhancing the work environment:

*With the complexity of the health care environment, the nursing workforce should be strengthened and empowered to enable them to fulfill their roles and enhance positive work environment.*

Another participant stated that:

*We need to improve the working environment including the PHC settings.*

Establishment of a database for the nursing workforce, enhancement of a balanced distribution of nurses in PHC sector were indicated, by some key informants, as important issues for effective planning for the nursing workforce:

*For effective nursing workforce planning, we need to establish a database for the nursing workforce and maintain a balanced distribution of nurses in the PHC sector to achieve UHC in Jordan.*

Ensuring a substantial amount of nurses, and building a critical mass of APNs, were also emphasized by the participants as factors which will enable nurses to improve the health of the population, and contribute to the achievement of UHC:

*Ensure enough supply of nurses and building a critical mass of APNs to meet current and future popu lation needs by securing solid education and practice programs.*

### Research

Issues that were addressed under the theme of research were: identify national priority research areas, support evidence-informed decision-making, measure the impact of advanced nurses’ role on PHC, measure the return on investments from nursing education and practice, and building capacity of nurses in research and evidence creation. They pointed out the need for establishing research programs which focus on priority areas which meet national health priorities and needs, as well as identify challenges at different practice levels and policy making processes:

*Nurses’ researchers need to conduct more research in order to investigate priority issues related to the role of nurses in attaining UHC and access to health.*

*More evidence-based research needs to be conducted to assess nurses’ awareness and contribution to attaining UHC, as well as the best practices to promote UHC.*

According to participants, solid research that provides evidence-informed policy and decisions can strengthen the health care system and nursing profession. Key informants also stressed the need for research studies that demonstrated the impact and cost effectiveness of APNs in strengthening the health care system, health outcomes, and meeting the national health needs including UHC:

*Nurses’ researchers can provide evidence about the cost effectiveness of universal health coverage and the effectiveness of health promotion interventions. Researchers should conduct studies about issues related to UHC.*

*Conduct evaluation research to investigate the readiness of graduate nurses to meet the national health changing needs.*

*…provide evidence that investment in APNs make a difference in health care.*

## Discussion

The results of this study showed congruency among key informants on most of the issues (policy and regulation, education, partnership, workforce, and research) that were raised to strengthen the role of APNs towards achievement of UHC. These issues are consistent with current nursing and midwifery strategies, reports and declarations that have emphasized the importance of nurses’ roles in achieving UHC, such as the WHO Strategic Directions for Nursing and Midwifery Development (SDNM) 2016-2020, WHO Global Forum for Government Chief Nursing and Midwifery Officers (WGFGCNO) in May 2014,Strengthening Nursing And Midwifery WHA64.7, Fifth TRIAD meeting in May 2014,Framework for Action Strengthening Nursing and Midwifery in the WHO EMRO 2015-2025 and the Jordanian National Nursing and Midwifery Strategy 2016-2025^7^ ^,^ ^19^ ^-^ ^20^.

It was evident that key informants in the present study recognized the need for a real transformation in the education and practice sectors in Jordan, in order to establish the groundwork for stronger nursing roles, including advancing practice nursing roles to meet the population practical health needs and the national health agenda. The transformation of nursing education and practice, including the expansion of the advanced role of nurses, are vital drivers for achieving UHC and the global health agenda^7^ ^,^ ^20^. In order to influence and deliver quality health care outcomes, there is a need not only to transform the way in which health care is provided, but also the way in which health care professionals are educated and trained. Evidence suggested a strong correlation between the level of education and the patients’ outcomes^21^ ^-^ ^22^.

Unfortunately, the education system in Jordan remains very traditional with major gaps, especially in the area of community, prevention, and PHC which are crucial for UHC^11^ ^,^ ^20^. This was the expected result of the fragmented silo efforts in improving nursing education and weak inter-professional and interdisciplinary collaboration among education, service, research and nursing institutions as well as lack of awareness of many nurse educators and leaders about contemporary health issues related to national and global health agenda. It is imperative that nursing emphasizes the wider health context, including its social determinants, financing, and sustainable development^7^ ^-^ ^8^ ^,^ ^10^ ^,^ ^23^.

Achievement of UHC in Jordan will place increased pressure on the nursing profession as a whole, and nursing educators and leaders in specific. Planning for an appropriate and well- prepared nursing workforce is becoming a major challenge in Jordan especially in light of the absence of a database for the nursing workforce. Establishment of a data base for the nursing workforce in Jordan is essential for improving the nursing workforce’s strategies and evidence- based workforce policies. This will ensure appropriate quantifications for the nursing workforce, demands and supply; appropriate geographical distribution and a balanced allocation of nurses in all health sectors including PHC, as well as better monitoring and alignment of investments in the nursing workforce with future needs and demands of the health systems to achieve the UHC and national health agenda^6^ ^-^ ^7^.

Nurses should find the right fit between their role and the demanding new global health agenda to ensure that the global health goal and its targets are achieved. Such balance requires the preparation of a critical mass of well-prepared nurses as well as the expansion of nursing roles and scope of practice. Therefore, major transformations in nursing education, standards, competencies, as well as an entire revision of the pedagogy and content, is required.

New nursing competencies are needed to ensure a productive nursing workforce and efficient health care system^7^ ^,^ ^11^ ^,^ ^19^.To assure achievement of UHC, the International Confederation of Midwives (ICM), International Council of Nurses (ICN) and WHO emphasized that “Ensuring nurses and midwives have the necessary competencies and scope of practice that allows them to effectively promote health and provide care is critical if we are to ensure equitable access to quality health services”^20^. As pointed out by key informants of the present study, nursing curricula should be sharpened by new solid competencies on leadership, policy making, policy dialogue, evidence based research, team work and collaboration, coupled with innovative teaching and learning strategies. Building capacity and capabilities of the nurses’ educators and faculty is crucial for understanding and implementation of a solid transformed nursing education programs^7^. Likewise, inter-professional education and collaboration are crucial for PHC and maximizing the dialogue about UHC to achieve the health agenda^7^. Therefore, regulatory, administrative and other barriers that limit health care providers from working together should be eliminated.

The land mark of the key informants was their persistence on advancing the role of the nurse, establishing continuous professional development programs and relicensing system as well as establishing a national board exam and residency programs for nurses to ensure a well-regulated nursing profession. This reflects a true professional maturity of nurse leaders’ vision for the quality of nursing workforce we need to prepare for the future to achieve UHC. Interestingly, with the expansion of the SDGS, and the global health agenda, there is a window of opportunity for the nursing profession to start developing nursing residency programs, especially in PHC. This also demands the strengthening of the education, practice, and work environment of the nursing workforce. In addition, strengthening the PHC sector and its workforce to be attractive for nursing students and nursing workforce is crucial for UHC^6^.

Advanced nursing practice in Jordan and the entire region is behind^7^ ^,^ ^10^. Unfortunately, and in spite of the increasing number of nurse specialists from the graduate nursing programs, nurses in Jordan are still not perceived as equal partners in health care. This might be due to the limited role and scope of practice of nurse specialists, as well as the absence of legal status of nurse specialists in Jordan. Another reason might be the increasing number of general physicians with the belief that there is no need to have other major players, such as nurses, in their “territory”. This is a profound misjudgment, especially with the fact that PHC in Jordan is totally disregarded, and devastated by lack of quantity and quality health care providers including physicians^11^ ^,^ ^13^ ^,^ ^20^. The challenging goals and targets of the SDGs implied a necessity for establishing a range of policy options to maximize the utilization of all health workforce, including the nursing workforce, who comprised about more than 70% of the health workforce, by investing in their full potential and scope of practice to contribute to the achievement of UHC and sustainable development^6^ ^,^ ^20^.

Nurse leaders identified the importance of eliminating the regulatory barriers that prevent nurses from practicing to the full extent of their knowledge and training, to achieve the UHC and health agenda. Advancing and expanding nurses’ roles and scopes of practice will not only result in producing and retaining competent nurses to meet population needs and UHC but also in maximizing the economic return on investment^7^ ^,^ ^10^. The Summary report on the regional nursing forum about the future of nursing and midwifery in the EMRO indicated that “Universal coverage is an opportunity to bridge the gap between access and coverage, coordinate increasingly complex care, fulfill nurses and midwives’ potential as primary care givers to the full extent of their education and training, enable the full economic value of contributions across care settings to be realized, and change the reference point from which nursing is understood”^10^.

The contribution of health employment and health workforce to enhancing global inclusive economic growth has been taken seriously by the UN. A High-Level Commission on Health Employment and Economic Growth was established by the Secretary-General of the United Nations in response to the United Nations general assembly resolution (A/70/L.32) in December 2015, recognizing that investing in new health workforce employment opportunities may add broader socio-economic value to the economy and contribute to the implementation of the 2030 Agenda for Sustainable Development^24^.

Nurses should invest in this momentum by directing their collective efforts and power to put in place regulatory frameworks for expanding nurses’ roles and scopes of practice; strengthening and empowering the nursing workforce and leadership through solid education, training, service, evidence based research and accreditation programs to ensure well-prepared, productive and influential nursing workforce overtime. We must be aware about the fact that nurses will only be able to influence and enhance the accessibility and quality of care as far as they have a legal status for their unique roles and strong leadership that support a wide scope of PHC responsibilities.

Policy and decision makers in the health sector must reconfirm their obligations for strengthening and empowering the nursing workforce and improving the work environment of the PHC, coupled with analyzing, evaluating and clearly defining roles for every group of PHC providers. It is impossible to design PHC without having clear roles for physicians, nurses, midwives, and other health care professionals. As a matter of fact, we need a clear role and scope of the advanced nursing practice to be communicated not only to physicians, but also to the public at large.

Such profound changes require a wider angle lens to capture the bigger picture which involves wide-reaching changes in policies of the health care system. These changes include better planning for the nursing workforce, enhancing a positive work environment; transforming education and practice; preparing a critical mass of qualified nurses including APNs; developing and enhancing the role of APNs; establishing solid regulatory mechanisms for nursing education and practice with the development of solid standards and competencies for the nursing profession; building capacity of practicing nurses and educators through solid training and continuing education programs; engagement of nurses in the policy and decision making process at all levels; identifying priority areas for nursing research agenda and creating evidence about nursing contributions to health outcomes and economic development, as well as fostering inter- professional and interdisciplinary partnerships.

The IOM report on the Future of Nursing emphasized the important contribution of nurses in “...building a health care system that will meet the demands for safe, quality, patient-centered, accessible, and affordable care”^25^. The main emphasis of the report was the importance of investment in the capabilities and skills of nurses to the full extent of their knowledge and training in addition to full partnerships with other health care professionals as indicated by the first powerful message in the IOM report: “Nurses should practice to the full extent of their education and training”^25^. It is imperative that nursing leadership is anchored within all mechanisms and sectors of the health care system^7^.Capacity building is highly needed for nurses on policy, leadership, regulation, education, practice and evidence based research to improve nursing outcomes and enhance their contribution to UHC and sustainable development. In fact, building the leadership and research capacity of nurses and nurse leaders becomes a strategic dimension of the utmost importance. Hence, strong nursing leadership in Jordan is crucial for strengthening nursing skills and competencies as well as enhancing the advanced practice nursing roles to promote the well-being of the population and achieve UHC.

To efficiently capitalize on the nursing workforce, to realize the global health goal and UHC, nurses should lead all interventions and programs that influence and transform their education, practice and care environments as well as lead serious efforts in creating evidence on the contribution that nurses are making towards UHC and economic development to ensure and sustain investments in nursing profession^7^ ^,^ ^20^ ^,^ ^24^.

Nurses as a whole should invest in their collective power to be recognized as evidence producers and largest group to create solutions for efficient health care delivery and sustainable health outcomes. Strong nursing leadership at all levels is key for realizing the promise of the nursing profession by providing evidence and proven strategies to inform policy and compact the nursing profession long standing challenges. “Nursing will be more valuable if it can demonstrate, by means of research, the effects of its interventions for achieving universal health coverage,”^25^.

High visibility of nursing leadership in policy dialogue, policy development and decision making, as well as evidence creation, will facilitate change in the mind set of health care providers and policy makers about the nursing workforce as integral members of the health care system reform. This requires a better understanding and learning of the ropes of policy and decision making including the legislative process^7^ ^,^ ^10^. This will enhance the process of transformation of nurses’ roles and scopes of practice as well as foster stronger inter-professional and multidisciplinary partnerships for effective collaborative efforts and outcomes to achieve UHC.

The challenging SDGs imposed on governments and policy makers to put in place supportive effective policies, actions and regulatory systems to eliminate all barriers hindering health professionals, including nurses from working to their full potentials to attain UHC and sustainable development^20^. Barriers include a lack of regulatory mechanisms, traditional education and practice system, ambiguous nursing role and limited scope of practice, lack of collaboration among health professional and heath sectors at all levels, lack of leadership, lack of nursing involvement in policy and decision making, unconducive work environment, and lack of decent work conditions and decent employment.

## Conclusion

Study findings confirmed the need to strengthen the role of APNs through a set of comprehensive strategies that take into consideration education, practice, policy and regulation, evidence based, workforce and work environment. Interestingly, this qualitative study has sensitized many key informants and nurse leaders about the power of UHC and SDGs, not only in enhancing the health, and well-being of the population but also in influencing the education and practice of health care professionals. Key informants and nurse leaders’ demand for strong awareness programs for nurses and other health care professionals about the UHC and SDGs realizing that profound changes in all nursing sectors should be aligned with the commitment of Jordan toward the UHC and SDGs.

They were all keen that Jordan needs to put in place solid policies and regulatory mechanisms that ensure a perfect fit and the relevance of nursing education, practice, research, and leadership skills to population needs that cater not only for current but also for future health needs.

A focus on UHC for the coming years could be a remarkable achievement for nursing education, practice, leadership and research at the national, regional, and global levels if nurses make true investments in this momentum to reaffirm their crucial roles that they need to play in achieving the health related goals and UHC of the post-2015 agenda. Nurses need to ensure thorough understanding and reflection of the relevance of their work to UHC principles and SDGs. UHC and sustainable development should be stemmed in nursing practice, education, leadership, and research as well as in building solid collaborative bridges within the nursing profession and with other health care professions, policy and decision makers.

We must realize that relying only on our good intentions, tradition, and past practices are no more acceptable for the nursing profession if we want to navigate the future of health care. Nurses in Jordan and all over the world, should demonstrate their unity and invest in their collective power to come up with solid specific nursing competencies, standards, and regulations to strengthen their position, not only as promoters of health but also as active actors in influencing policy and health dialogue on UHC and sustainable development.

Ambitious goals and targets are often needed to inspire and accelerate progress. Nurses have incredible potential to play a significant role in achieving the very challenging health agenda and UHC. The main issue will be how serious and capable we are in evaluating and measuring the return on investment from investments in the nursing workforce (including education, skills, advance nursing practice, leadership and evidenced based research) to contribute to the achievement of UHC and enhancement of economic growth.
